# From prevalent to personal: how social exposure predicts attitudes toward non-suicidal self-injury and what prevalence reveals

**DOI:** 10.3389/fpsyg.2025.1652207

**Published:** 2025-11-10

**Authors:** Rosie James, Elin Lundgren, Daiva Daukantaité, Magnus Nilsson

**Affiliations:** 1 Department of Clinical Sciences Malmö, Lund University, Malmö, Sweden; 2 Department of Psychiatry, Skåne University Hospital, Lund, Sweden; 3 Department of Psychology, Lund University, Lund, Sweden

**Keywords:** non-suicidal self-injury, stigma, attitudes, university student, social exposure, prevalence, intergroup contact

## Abstract

**Introduction:**

Non-suicidal self-injury (NSSI) is often met with stigma, which can deter individuals from seeking help. While most research on NSSI stigma has focused on clinical settings, the attitudes of those most likely to receive an NSSI disclosure, such as peers, friends, and family, remain underexplored. Building on qualitative findings that positive disclosure experiences can foster supportiveness, this study aimed to quantitatively examine how social exposure, gender, personal experience with NSSI, and mental health relate to supportive attitudes toward NSSI within social circles. We also assessed the prevalence and awareness of NSSI in close relationships, among university students.

**Method:**

A total of 1,430 Swedish university students completed a baseline survey, with 721 participating in a six-month follow-up. Measures included self-reported NSSI and mental health history, awareness of NSSI in others (social exposure), and attitudes toward NSSI.

**Results:**

Correlational and regression analyses showed all predictors were significantly associated with attitudes, with personal NSSI experience emerging as the strongest. Paired samples t-tests revealed a small but significant increase in supportive attitudes among participants who reported new social exposure at follow-up (*n* = 67), with no change observed among those without new exposure. Lifetime prevalence of NSSI in the sample was estimated at 38.7%, and 62.5% reported social exposure.

**Discussion:**

These findings suggest that while personal experience with NSSI is the strongest predictor of supportive attitudes, increased social exposure may serve as an ethically modifiable factor in stigma reduction. Given the high prevalence and social visibility of NSSI in university populations, these settings may offer valuable opportunities for targeted anti-stigma initiatives.

## 1 Introduction

Poor mental health among students is a globally recognized concern (Whitlock et al., 2011; Bruffaerts et al., 2018; Ebert et al., 2019; Sheldon et al., 2021), with one in four undergraduates experiencing depression and one in six reporting suicidal ideation (Sheldon et al., 2021). One particularly concerning behavior is non-suicidal self-injury (NSSI), defined as deliberate self-inflicted harm (e.g., cutting, burning, carving) without the intention to die (Nock and Favazza, 2009). National reports from Sweden reflect these concerns, with rising rates of self-harm (Folkhälsomyndigheten, 2023) and psychological distress (CSN, 2020) reported among young people and university students. While most common in adolescence (Plener et al., 2015), NSSI shows a second prevalence peak around age 20 (Gandhi et al., 2018), which coincides with the typical age of university students. Among this group, lifetime prevalence rates have been reported between 20.4–39% (Cerutti et al., 2012; Cipriano et al., 2017; Sivertsen et al., 2019; Kiekens et al., 2023a).

Emerging evidence suggests that rates of hospitalization due to NSSI, which often reflects more severe forms of NSSI, have increased in recent years potentially exacerbated by the COVID-19 pandemic (Madigan et al., 2023; Roumeliotis et al., 2024). Given its association with severe mental illness and increased suicide risk (Whitlock et al., 2013; Fox et al., 2015; Ribeiro et al., 2016), seeking support for NSSI is critical, but stigma and shame often serve as significant barriers to help-seeking (Rosenrot and Lewis, 2020; Simone and Hamza, 2020; Mirichlis et al., 2022). Since help-seeking often begins with disclosing NSSI to someone close, such as a friend, partner, or family member (Armiento et al., 2014; Ammerman, 2018; Simone and Hamza, 2020), these interpersonal disclosures represent a key first step in the broader help-seeking process. Supportive responses to such disclosures have been shown to increase compassion in those receiving the disclosure and facilitate further help-seeking in those with NSSI (Toste and Heath, 2010; Park and Ammerman, 2020).

Recipients of NSSI disclosures, typically friends or family, are referred to here as individuals with *social exposure* to NSSI. Gaining insight into their attitudes is essential for developing strategies that foster more supportive environments and ultimately reduce the stigma surrounding NSSI. Existing literature on healthcare professionals’ attitudes, shows that greater knowledge of, and experience working with, individuals with NSSI is associated with reduced stigma and, correspondingly, more supportive attitudes (Muehlenkamp et al., 2013; Pintar Babič et al., 2020). Research on those with social exposure to NSSI remains largely limited to small-scale, qualitative studies, which, while valuable, often lack generalizability. Qualitative findings, nevertheless, offer preliminary insights. They suggest that NSSI disclosures can encourage increased sympathy, awareness, and understanding of the emotional and psychological motivations behind the behavior among peers (Law et al., 2009; Fisher et al., 2017; Nielsen and Townsend, 2018; Gayfer et al., 2020; Simone et al., 2023). A narrative review also hints at the potential stigma-reducing benefits of NSSI disclosures, suggesting that social-contact-based interventions are the most effective for improving attitudes and stigma-related knowledge in the public (Thornicroft et al., 2016).

These findings align with Intergroup Contact theory, which suggests that meaningful interactions between members of different social groups can reduce prejudice and stigma, particularly under conditions of equal status, cooperation, and institutional support (Allport, 1954; Tajfel and Turner, 1979). More recent developments have expanded the theory to emphasize the role of affective processes, such as empathy and reduced anxiety, in shaping intergroup attitudes (Tropp et al., 2016). The theory also recognizes the impact of indirect contact, such as knowing someone who has a close relationship with an outgroup member, which can similarly improve attitudes (Wright et al., 1997). In the context of NSSI, disclosure may function as a form of social exposure that mirrors intergroup contact, allowing individuals without personal experience of NSSI to engage empathetically with those who do.

This mechanism has been studied in relation to mental illness stigma reduction campaigns but there is a tendency to omit focus and support for addressing NSSI specifically (Banerjee and Meheli, 2022) as well as concerns about the lack of public resources available (Toste and Heath, 2010). Omission of NSSI from such initiatives likely stems from fears of behavioral contagion (Conigliaro and Ward-Ciesielski, 2023) and the increased emotional burden and uncertainty placed on those receiving NSSI disclosures (Fisher et al., 2017). While these concerns are valid, research also suggests that social exposure to NSSI can prompt help-seeking among those receiving NSSI disclosures if they need support themselves (Li et al., 2024). Social exposure to NSSI therefore presents both challenges and opportunities: while it may expose individuals to emotional strain if coupled with an absence of adequate support, it can also yield benefits for both parties when appropriate resources are available, including increased awareness, reduced stigma, and improved help-seeking behaviors. It is therefore essential to investigate how attitudes toward NSSI are shaped, examining how social exposure affects these attitudes, and whether changes in exposure can foster more supportive, less stigmatizing attitudes as the qualitative literature and theory suggests.

To our knowledge, no large-scale quantitative studies have investigated the factors influencing public attitudes toward NSSI, particularly among those with social exposure to it. This study therefore addresses a gap in quantitative research on public attitudes toward NSSI by examining predictors of supportive attitudes, with a particular focus on the role of social exposure. Grounded in Intergroup Contact theory, this study investigates whether social exposure to individuals who engage in NSSI is associated with more supportive attitudes. Social exposure is of particular theoretical interest, as it reflects a form of intergroup contact that is both ethically modifiable and potentially impactful in shaping public perceptions.

The study therefore has three primary aims. First, we examine whether supportive attitudes toward NSSI are associated with gender, personal experience with NSSI, mental health problems, and social exposure to NSSI. Second, we assess the relative strength of these variables, evaluating whether social exposure stands out as a key factor in line with Intergroup Contact theory. Third, we explore whether acquiring new social exposure to NSSI over time is associated with corresponding changes in attitudes, thereby exploring the dynamic potential of contact-based interventions. As a secondary aim, we also provide updated prevalence estimates for social exposure to NSSI, lifetime engagement with NSSI, and mental health problems, to contextualize our findings and contribute to the broader literature.

## 2 Materials and methods

### 2.1 Study design and setting

The study uses a mixed design, combining cross-sectional and short-term longitudinal elements. Two rounds of self-report questionnaires were sent out via email to all students registered during that academic term at a large university in Southern Sweden, first in February 2022 and then again in September 2022. While baseline data were used for cross-sectional analyses, a subset of participants completed both surveys, enabling longitudinal examination of attitudes toward NSSI over time. The study was approved by the Swedish Ethics Review Authority (Dnr 2021–05102), where “Dnr” refers to the official registration number of the approval decision. No additional university-level approval was required. Participation was voluntary, without compensation, and digital informed consent was obtained from all participants at both time points. Unique anonymized codes ensured confidentiality while enabling baseline-follow-up matching.

### 2.2 Participants

Following the survey distribution via email sent out to all Lund University students, as part of a broader data collection effort, the baseline survey yielded a voluntary response sample of 1,500 responses, which was reduced to 1,430 after data cleaning [mean (SD) age 26 (7.33); 68.6% female]. Participants were excluded if they answered incorrectly on any control question or exhibited “straightlining” (i.e., repetitive responses across measures), indicating inattentive responding (Kim et al., 2019). At the six-month follow-up, 721 participants [mean (SD) age 26.3 (8.00); 68.4% female] completed the same questionnaire. To enhance participation across both data collection periods, a follow-up reminder email was sent (Manzo and Burke, 2012). Table 1 provides a detailed breakdown of baseline characteristics and prevalence estimates by gender.

**Table 1:** Baseline characteristics of participants by gender.

Baseline characteristic	Total (*N* = 1,430)	Women (*N* = 981)	Men (*N* = 400)	Other (*N* = 36)	Test statistic
Age, mean (SD)	26.21 (7.32)	26.29 (7.33)	26.21 (7.56)	24.64 (5.16)	*F* = 0.818
Lifetime NSSI prevalence, *n* (%)	554 (38.7)	436 (44.4)_c_	85 (21.3)_c_	26 (72.2)_c_	*χ^2^* = 83.27^**^
Lifetime mental health problems, *n* (%)	1,172 (82.0)	827 (84.3)_c_	297 (74.3)	36 (100.0)_c_	*χ^2^* = 28.58^**^
Social exposure to NSSI, *n* (%)	894 (62.5)	648 (66.1)_c_	209 (52.3)_c,d_	29 (80.6)_c_	*χ^2^* = 28.24^**^
Above depression cutoff, *n* (%)	330 (23.1)	235 (24.0)	78 (19.5)	12 (33.3)	*χ^2^* = 7.18
Above anxiety cutoff, *n* (%)	456 (31.9)	333 (33.9)_c_	100 (25.0)_c,d_	18 (50.0)_c_	*χ^2^* = 16.35^**^

Depression indicator measured by the SCL-CD6 and anxiety indicator measured by the GAD7. *Post-hoc* comparisons using a Bonferroni correction are reported in subscript letters where use of the same letter indicates a significant difference (*p* < 0.05) between the genders in each factor. ^**^p < 0.001.

### 2.3 Measurements

#### 2.3.1 Attitudes toward NSSI

The valence of attitudes toward individuals who engage in NSSI was measured using the Lund Tolerance toward Self-Harm (LUTOSH) scale, which includes five statements (Nilsson et al., 2020), for example, “People who self-harm should not be blamed.” Responses were rated on a 10-point Likert scale from 1 (*completely disagree*) to 10 (*completely agree*), yielding total scores ranging from 5 to 50. Items 3 and 4 were reverse-coded, and higher scores indicated more supportive attitudes. The LUTOSH scale has demonstrated convergent validity and moderate internal consistency (Cronbach’s *α* = 0.72) in Swedish populations (Nilsson et al., 2020). In this study, Cronbach’s α was 0.66. To our knowledge, LUTOSH is the only scale specifically designed to measure public attitudes toward those who self-harm.

#### 2.3.2 Social exposure to NSSI

Participants were asked whether they were aware of a friend or family member who engages in NSSI, with a binary response option 0 (*no*) and 1 (*yes*).

#### 2.3.3 Lifetime prevalence of NSSI and mental health problems

Participants were asked two separate questions about their personal experience with NSSI and mental health problems. They reported the recency of these experiences using four response options: “Earlier in life, but not during the last six months,” “Within the last six months but not before,” “Both within the last six months and earlier,” and “Neither within the last six months nor before.” For the lifetime prevalence calculation and the variable personal experience with NSSI, responses were dichotomized 0 (*no personal experience*) and 1 (*personal experience*).

#### 2.3.4 Depressive and anxiety symptoms

Depressive symptoms were assessed using the Swedish version of the Symptom Checklist - Core Depression (SCL-CD6), which includes items such as, “feeling blue/sad” and “blaming yourself” (Magnusson Hanson et al., 2014). Participants rated how much they were affected by these feelings on a 5-point Likert scale ranging from 1 (*not at all*) to 5 (*extremely*). A cutoff of ≥17 indicated probable clinical depression (Magnusson Hanson et al., 2014). The measure has shown good internal reliability, and Cronbach’s *α* for the current sample was 0.90.

Anxiety symptoms were measured using the Swedish version of the Generalized Anxiety Disorder-7 (GAD-7) anxiety scale, which includes seven items assessing the frequency of symptoms over the last 2 weeks (Spitzer et al., 2006). Responses were recorded on a 4-point Likert scale ranging from 0 (*not at all*) to 3 (*daily*). Example items include “feeling nervous, anxious and on edge.” Although widely used in Sweden, the translated scale has not been validation in its present form (Ahlqvist Lindqvist et al., 2021). Cronbach’s α for the current sample was 0.91.

### 2.4 Statistical analyses

All analyses were conducted using IBM SPSS Statistics 28.0 (IBM Corp, 2021). Attrition between baseline and follow-up was assessed using independent samples t-tests for continuous variables and Chi-square tests for categorical variables. Associations between all variables were examined using bivariate correlations, and a multiple linear regression model was used to identify the relative strength of predictors of attitude toward NSSI. Assumptions for regression were met and forced-entry method was used as no single predictor was expected to be more influential than the others.

For the longitudinal analysis, two subsets were identified based on changes in responses to whether participants knew friends or family who engaged in NSSI. Paired samples t-tests were used to compare mean attitude scores among those who reported new social exposure at follow-up, and among those who did not, to assess within-person changes and control for general shifts in attitudes. Finally, descriptive analyses were used to examine the lifetime prevalence of NSSI, mental health problems, and rates of social exposure to NSSI across gender groups in the baseline sample. Where applicable, scales were categorized using established cutoff values.

## 3 Results

### 3.1 Attrition analysis

Follow-up participants (*n* = 721) were compared to non-responders (*n* = 709) across all measured baseline variables. Participants with a history of NSSI were significantly more likely to respond to the follow-up (53.8%) than those without (48.3%), *χ^2^*(1) = 4.11, *p* < 0.05. However, the small effect size (Cramer’s *V* = 0.054) indicates a limited practical significance. No significant differences were found for baseline attitudes toward NSSI, depression, anxiety, social exposure to NSSI, and personal experience of mental health problems, nor were they found for gender or age.

### 3.2 Correlation analyses

To address the first primary aim, Pearson’s correlation analyses (see Table 2) examined relationships between LUTOSH scores and key variables: gender (0 = male, 1 = female), lifetime prevalence of NSSI (0 = no, 1 = yes), lifetime prevalence of mental health problems (0 = no, 1 = yes) and social exposure to NSSI (0 = no, 1 = yes). Significant positive correlations were found between supportive attitudes and being female (*r* = 0.25, *p* < 0.001), having engaged in NSSI at some point (*r* = 0.32, *p* < 0.001), having had mental health problems at some point (*r* = 0.25, *p* < 0.001), and social exposure to NSSI (*r* = 0.17, *p* < 0.001).

**Table 2:** Pearson’s correlation matrix for attitudes toward NSSI, gender, lifetime NSSI prevalence, social exposure, and lifetime mental health problem prevalence.

Variable	n	M	SD	1	2	3	4	5
1. LUTOSH total^a^	1,430	38.17	8.58	-				
2. Gender^b^	1,381	0.71	0.45	0.25^**^	-			
3. Lifetime NSSI prevalence^c^	1,430	0.39	0.49	0.32^**^	0.22^**^	-		
4. Lifetime mental health problem prevalence^c^	1,430	0.82	0.39	0.25^**^	0.12^**^	0.33^**^	-	
5. Social exposure to NSSI^c^	1,430	0.63	0.48	0.17^**^	0.13^**^	0.20^**^	0.07^*^	-

a Values represent the average LUTOSH score at baseline.

b 0 = male and 1 = female.

c 0 = no and 1 = yes.

^**^p < 0.001. ^*^p < 0.01.

### 3.3 Regression analyses

In line with the second primary aim, a multiple linear regression analysis examined the predictive power of the aforementioned variables on attitudes toward NSSI. The model explained 16.1% of the variance in attitude scores, *F*(4,1,376) = 65.94, *p* < 0.001, *R*^2^ = 0.161. All four variables significantly predicted more supportive attitudes (see Table 3), with lifetime prevalence of NSSI being the strongest (*β* = 0.21, *p* < 0.001) compared to lifetime prevalence of mental health problems (*β* = 0.15, *p* < 0.001) and being female (*β* = 0.18, *p* < 0.001). Social exposure to NSSI was also a significant predictor of supportive attitudes, though its effect size was smaller than other predictors (*β* = 0.09, *p* < 0.001).

**Table 3:** Linear model of predictors of attitudes toward NSSI.

Predictor	B	SE B	95% CI		*β*	*t*	*p*
			LL	UL			
Constant	30.60	0.61	29.41	31.80	-	50.32	< 0.001
Gender^a^	3.41	0.48	2.47	4.36	0.18	7.09	< 0.001
Lifetime NSSI prevalence^b^	3.72	0.48	2.78	4.65	0.21	7.78	< 0.001
Lifetime mental health problem prevalence^b^	3.26	0.58	2.13	4.39	0.15	5.65	< 0.001
Social exposure to NSSI^b^	1.50	0.45	0.62	2.37	0.09	3.35	< 0.001

*F*(4,1,376) = 65.94, *p* < 0.001, Adjusted R^2^ = 0.16. Dependent variable: LUTOSH total. Total N = 1,430. CI, Confidence Interval; LL, Lower Limit; and UL, Upper Limit.

a 0 = male and 1 = female.

b 0 = no and 1 = yes.

### 3.4 Paired-samples t-tests

In line with the third primary aim, a paired samples t-test was conducted to examine whether new social exposure to NSSI was associated with changes in attitudes after 6 months. Participants who reported new social exposure to NSSI at follow-up (*n* = 67) showed a significant increase in positive attitudes from baseline (M = 37.76, SD = 7.47) to follow-up (M = 39.16, SD = 7.42), *t*(66) = 2.06, *p* = 0.043 (two-tailed), with a small effect size (Cohen’s *d* = 0.25). In contrast, among participants who reported no new social exposure at follow-up (*n* = 654), there was no significant change in attitudes between baseline (M = 38.62, SD = 8.22) and follow-up (M = 38.43, SD = 8.41), *t*(653) = −0.70, *p* = 0.484 (two-tailed). The two groups did not show any significant differences in baseline attitudes toward NSSI (*t*(719) = 0.82, *p* = 0.413), age (*t*(719) = −0.67, *p* = 0.946), gender distribution (χ^2^(1) = 1.03, *p* = 0.311), personal experience with mental health problems (χ^2^(1) = 0.74, *p* = 0.391), or personal experience with NSSI (χ^2^(1) = 0.12, *p* = 0.733).

### 3.5 Prevalence estimates

In line with the secondary aim, prevalence statistics and gender analyses are reported in Table 1. Lifetime NSSI prevalence was 38.7%. Significantly fewer men (21.3%) reported lifetime NSSI than women (44.4%) or Other (72.2%), *χ^2^*(3) = 83.27, *p* < 0.001. Social exposure to NSSI was reported by 62.5% of the population, with significantly fewer men (52.3%) reporting this than women (66.1%) or Other (80.5%), *χ^2^*(3) = 28.24, *p* < 0.001. Mental health problems were reported by 82% of the sample, with significantly fewer men (74.3%) reporting these problems than women (84.3%) or Other (100%), *χ^2^*(3) = 28.58, *p* < 0.001. Women and individuals reporting Other as their gender were also significantly more likely to exceed the severe anxiety cutoff than men, *χ^2^*(3) = 16.35, *p* < 0.001. No significant differences were found between the genders and whether the depression scores were above or below the clinical cutoff or the frequency of NSSI within the last 6 months.

## 4 Discussion

This study examined how gender, personal experience with NSSI, mental health problems, and social exposure to NSSI relate to attitudes toward individuals who self-injure, and whether acquiring new social exposure over time relates to any changes in these attitudes. Using a large sample of university students, we found that all four factors significantly predicted more supportive attitudes, with personal experience with NSSI emerging as the strongest predictor. Importantly, participants who reported new social exposure at follow-up showed a small but significant increase in supportiveness, suggesting that interpersonal contact may foster attitudinal change. These findings support the relevance of Intergroup Contact Theory in the context of NSSI and highlight social exposure as a potentially modifiable factor in stigma reduction. Given the high prevalence of NSSI and social exposure within university populations, these results underscore the importance of targeted anti-stigma initiatives in educational settings.

The following sections explore these relationships in greater detail, beginning with the role of personal experience and gender in shaping attitudes toward NSSI.

### 4.1 The relationship between personal experience, gender and attitudes

The strongest predictor of supportive attitudes was personal experience with NSSI, which likely reflects cognitive empathy and experiential knowledge (Maunder and White, 2019). Individuals with a history of mental health problems also exhibited greater supportiveness, even if they had not engaged in NSSI themselves. This suggests that broader mental health struggles may cultivate empathy toward those engaging in NSSI, and not just those with the same experiences. Previous research supports this, but our results demonstrate its specific effect on public attitudes toward NSSI (Stuber et al., 2014).

Gender differences were also observed, with women demonstrating more supportive attitudes toward NSSI than men, which aligns with societal expectations in Western cultures that encourage caregiving roles and emotional expression among women (Chaplin, 2015; Eagly and Koenig, 2021). These findings are in line with prior research (Muehlenkamp et al., 2013; Pintar Babič et al., 2020).

### 4.2 The relationship between social exposure to NSSI and attitudes

Social exposure to NSSI has previously been linked to increased empathy and understanding in qualitative studies (Gayfer et al., 2020; Simone et al., 2023). This study is the first to demonstrate this relationship using large-scale, quantitative data. Initially, we observed a weak but significant correlation between social exposure and more supportive attitudes. Furthermore, having social exposure to NSSI significantly predicted more supportive attitude scores. While it was not the strongest predictor in our regression model, it is notably the only factor in this study that could be ethically influenced by stigma-reduction efforts.

We therefore further explored this factor by comparing within-person attitude changes, which indicated that participants who acquired new social exposure to NSSI after 6 months demonstrated a small but significant increase in supportive attitudes. Those who did not report changes in social exposure did not demonstrate any significant changes in attitudes. Gender, age and personal experience did not differ between these groups at baseline implying that their influence was likely minimal. This comparison strengthens the argument that the observed attitude shift is associated with the acquisition of social exposure to NSSI, rather than general changes over time. Although the observed shift in attitudes was small, even modest changes can be meaningful, especially when they accumulate across larger populations or are integrated into broader stigma-reduction initiatives (Götz et al., 2022).

Together, these findings tentatively support the idea that acquiring social exposure to NSSI may contribute to supportive shifts in attitudes toward those that engage in it. One interpretation of this finding is that social exposure may reduce stigma by normalizing the presence of NSSI as a coping strategy that warrants support, rather than judgment. Importantly, this does not imply endorsement of the behavior, but rather an increased understanding of its psychological underpinnings which reflects the attitudes measured by the scale we have used (Nilsson et al., 2020). This aligns with the Intergroup Contact Theory of stigma (Allport, 1954; Tajfel and Turner, 1979), which holds that interaction with stigmatized groups can reduce prejudice. This theory has been applied in the *College Toolbox Project*, a North American university initiative aimed at reducing mental illness stigma by encouraging interaction between affected and unaffected students. The project led to decreased stigmatizing attitudes and a more inclusive campus climate (Pescosolido et al., 2020; Manago and Krendl, 2023). While the project did not focus on NSSI, it illustrates the relevance of the underlying principle.

The *Signs of Self-Injury* program applied similar ideas to adolescent NSSI, training students to recognize signs in peers, show concern, and involve a trusted adult (Muehlenkamp et al., 2009). The intervention improved knowledge and help-seeking attitudes, in this structured setting and considered the unique needs of minors. The study additionally explored concerns around behavioral contagion following increased exposure to NSSI, which is often cited as a potential risk (Conigliaro and Ward-Ciesielski, 2023). However, they found no evidence of iatrogenic effects resulting from their initiative (Muehlenkamp et al., 2009). This highlights the importance of contextual factors, specifically the presence of appropriate support structures. Without these, disclosures can leave recipients feeling overwhelmed, particularly when they feel ill-equipped to respond (Fisher et al., 2017; Simone et al., 2023). However, with the right resources in place, disclosure can foster increased empathy, understanding, and support, while reducing the risk of distress. This underscores the need for initiatives that not only encourage safe disclosure but also ensure that recipients are prepared and supported, as demonstrated by programs like the College Toolbox Project and the Signs of Self-Injury initiative. Equipping individuals to respond effectively helps amplify the positive outcomes of disclosure while mitigating the risks of behavioral contagion.

### 4.3 Prevalence of NSSI, social exposure, and mental health problems

Given the impact of social exposure on attitudes, understanding the broader prevalence of NSSI and related mental health problems is essential for contextualizing these findings. In our sample, 62.5% of participants reported social exposure to NSSI. Women and Other reported social exposure more frequently than men, possibly reflecting both higher NSSI in these groups and a greater likelihood of disclosure within the same gender. A better understanding of how disclosure recipients understand stigma and influence help-seeking could strengthen future interventions. Our findings suggest that university settings provide a particularly valuable context for such initiatives.

The lifetime prevalence of NSSI in our sample was 38.7%, closely aligning with one prior study reporting 39% (Cerutti et al., 2012). However, NSSI prevalence estimates vary widely due to methodological differences (Cipriano et al., 2017), making it challenging to place the present findings in the context of existing figures. For instance, our findings contrast greatly with a recent international meta-analysis, which reported a 20% lifetime prevalence among first-year university students (Kiekens et al., 2023b). These discrepancies may stem from regional variations, as the meta-analysis did not include Swedish universities, or differences in measurement approaches.

In contrast, our prevalence estimates for those with likely depression (one in four) and generalized anxiety (one in three) are consistent with global university student estimates (Sheldon et al., 2021), Swedish national data (Folkhälsomyndigheten, 2023), and European regional data (Li et al., 2022).

### 4.4 Application of results

The prevalence rates observed underscore the growing mental health crisis among university students and highlight the need for targeted interventions. Given the high proportion of individuals in the current population who report social exposure to NSSI, universities are uniquely positioned to play a pivotal role in shaping intervention and awareness strategies. This raises the question of how such exposure can be harnessed to reduce stigma. By leveraging the findings on the predictors of supportive attitudes toward NSSI, universities can implement programs that foster understanding, empathy, and help-seeking.

Swedish universities already have dedicated teams supporting student mental health, providing an ideal foundation for integrating such a program (Stockholms universitet, 2024). Sweden’s Public Health Authority (Folkhälsomyndigheten) recently launched an initiative to improve young people’s mental health through online support and information (Folkhälsomyndigheten, 2024), yet it has largely overlooked NSSI. Given the high prevalence of NSSI among university students, incorporating NSSI into existing initiatives could strengthen support systems and reduce stigma, using the principles highlighted in this study. The current absence of NSSI-specific resources may inadvertently reinforce stigma and limit available support. Applying our findings within this framework presents an opportunity to create a more inclusive and supportive environment for the many students affected by NSSI.

### 4.5 Strengths, limitations, and further research

This study offers several strengths, including its novel contributions to the research field, a robust quantitative design, and a university-wide approach that enhances its relevance to student populations. The large sample size strengthens the reliability of the findings, despite a relatively low response rate of 4.7%, which was expected considering the large, non-compensated, and online design (Cho et al., 2013). The gender distribution in our sample, with 68% female participants, generally reflects the broader demographic composition of the university. According to Lund University’s 2021 annual report, 57% of students were women (Lund University, 2022). This supports the representativeness of our sample; however, gender-related selection bias cannot be ruled out, which is common in survey-based research.

Additional limitations include the sample’s homogeneity in age, which may restrict generalizability to older populations. The internal consistency of the LUTOSH scale was somewhat low (*α* = 0.66), potentially affecting the reliability of attitude measurements. While all predictors were statistically significant, effect sizes were modest. These small effects may still be meaningful when scales across larger populations due to potential cumulative effects (Pescosolido et al., 2020; Götz et al., 2022). Furthermore, some participants may have underreported social exposure at baseline due to recall bias, which could have influenced longitudinal comparisons. Finally, as this study is observational, causal interpretations cannot be made. Future research should explore these relationships using experimental or longitudinal designs and consider qualitative follow-ups to better understand the mechanisms behind attitude change.

## 5 Conclusion

This study provides insights into attitudes toward non-suicidal self-injury (NSSI), with personal experience with NSSI being observed as the strongest predictor of more supportive attitudes. Being female, having experience of mental health problems and social exposure were also significant predictors, however the latter presents a unique opportunity to influence anti-stigma efforts as it is a factor that could be encouraged without significant ethical issues. Social exposure (i.e., awareness of friends or family who self-injure) can come about from promoting NSSI disclosures, a phenomenon often omitted from anti-stigma mental health campaigns. Our findings suggest that due to the high prevalence of NSSI and high social exposure to NSSI in university populations, universities are an ideal target for implementation of such initiatives. This widespread presence within social networks reinforces the potential of interpersonal connections to shape stigma reduction and encourage help-seeking behaviors. While awareness campaigns promoting social connectedness are discussed, it is equally crucial to provide adequate support for individuals receiving NSSI disclosures. Our findings and subsequent discussion underscore the need for enhanced education and awareness initiatives at universities that focus on NSSI, promote social connectedness and equip individuals with the resources to provide meaningful support.

## Data Availability

Due to data sensitivity, access to raw data will be granted upon reasonable request following ethical approval update.
